# Premature mortality 16 years after emergency department presentation among homeless and at risk of homelessness adults: a retrospective longitudinal cohort study

**DOI:** 10.1093/ije/dyad006

**Published:** 2023-02-08

**Authors:** Rachel Zordan, Jessica L Mackelprang, Jennie Hutton, Gaye Moore, Vijaya Sundararajan

**Affiliations:** Inclusive Health, St Vincent’s Hospital, Melbourne, Victoria Parade, Fitzroy, Victoria 3065, Australia; Melbourne Medical School, Faculty of Medicine, Dentistry and Health Sciences, University of Melbourne, Fitzroy, Victoria 3065, Australia; Department of Psychological Sciences, School of Health Sciences, Swinburne University of Technology, Melbourne, Victoria 3122, Australia; Melbourne Medical School, Faculty of Medicine, Dentistry and Health Sciences, University of Melbourne, Fitzroy, Victoria 3065, Australia; Emergency Department, St Vincent's Hospital Melbourne, Melbourne, Victoria 3065, Australia; The Victorian Virtual Emergency Department, The Northern Hospital, Epping, Victoria 3076, Australia; Inclusive Health, St Vincent’s Hospital, Melbourne, Victoria Parade, Fitzroy, Victoria 3065, Australia; Inclusive Health, St Vincent’s Hospital, Melbourne, Victoria Parade, Fitzroy, Victoria 3065, Australia; Melbourne Medical School, Faculty of Medicine, Dentistry and Health Sciences, University of Melbourne, Fitzroy, Victoria 3065, Australia

**Keywords:** Homelessness, at risk of homelessness, mortality, cohort study, cause-specific

## Abstract

**Background:**

People experiencing homelessness have an increased risk of mortality. The association between being at risk of homelessness and premature mortality is unclear. We aimed to determine all-cause and cause-specific mortality in patients who were homeless, at risk of homelessness (marginally housed), or housed.

**Methods:**

This retrospective longitudinal cohort study compared mortality patterns in adult patients identified in 2003/04 by linking data from an Australian metropolitan emergency department to national mortality data. We used Cox proportional hazards models to estimate associations between housing status and mortality. To address competing risks, cause-specific hazards were modelled and transformed into stacked cumulative incidence functions.

**Findings:**

Data from 6290 patients (homeless deceased = 382/1050, marginally housed deceased = 259/518, housed deceased = 1204/4722) found increased risk of mortality in homeless [hazard ratio (HR) = 4.0, 95% confidence interval (CI) = 2.0–3.3) and marginally housed (HR = 2.6, 95% CI = 3.4–4.8) patients. Homeless patients had an excess risk from external causes (HR = 6.1, 95% CI = 4.47–8.35), cardiovascular disease (HR = 4.9, 95% CI = 2.78–8.70) and cancer (HR = 1.5, 95% CI = 1.15–2.09). Marginally housed patients had increased risk from external causes (HR = 3.6, 95% CI = 2.36–5.40) and respiratory diseases (HR = 4.7, 95% CI = 1.82–12.05). Taking account of competing risk, marked inequality was observed, with homeless, marginally housed and housed patients having probabilities of death by 55 years of 0.2, 0.1 and 0.02, respectively.

**Conclusions:**

Mortality rates were elevated in patients who were homeless or at risk of homelessness. Increasing numbers of people are at risk of homelessness, and the effect of this on mortality is relatively unrecognized. Marginal housing may assuage some risk of premature mortality associated with homelessness; however, it is not equivalent to stable housing.

Key Messages

- This study tests the associations of experiencing homelessness or being at risk of homelessness (marginally housed) with mortality, using patient data linked to a national mortality database.
- Both the homeless and the at risk of homelessness experiences were associated with an increased risk of mortality.
- Detailing all-cause and cause-specific mortality in patients who are at risk of homelessness fills a gap in the literature and is a novel contribution.
- Marginal housing may lessen some risk of premature mortality associated with homelessness; however, it is not equivalent to appropriate, stable housing.

## Introduction

The prevalence of homelessness is increasing in most high-income countries.^1^ The risk of premature mortality in homeless populations ranges from two to 10 times that of housed patients^2^ and the general population,^3^ respectively. External causes^4–7^ including alcohol^8^ and drugs,^8^^,^^9^ cancer^5^ and cardiovascular disease (CVD)^4^^,^^9^ are the leading causes of death among people who experience homelessness. Homelessness is frequently thought of as rough sleeping, which is the extreme and visible end of the homelessness continuum. However, homelessness is more complex and is often obscured in rooming houses, motels or vehicles. This more prevalent type of homelessness is also associated with premature mortality.^10^

Marginal housing is rented accommodation, below minimum standards, associated with government support and frequently tenuous. People in marginal housing are at risk of homelessness.^11^ The relationship between marginal housing and ill health is documented.^12^ However, little is understood about the association between marginal housing and premature mortality. Two studies investigated mortality among people residing in marginal housing^2^^,^^13^ but they produced conflicting findings, leaving uncertain the role of marginal housing in mortality. Further, they were limited by a small number of cases^13^ and the analysis of in-hospital deaths exclusively.^2^ To address this gap in the literature, we compared mortality patterns among homeless, marginally housed and housed adults by linking patient data from a metropolitan emergency department (ED) in Melbourne, Australia, to national mortality data.

## Methods

This retrospective, longitudinal, cohort study analysed data from patients attending an ED in a 540-bed, inner-city, tertiary hospital between 1 January 2003 and 31 December 2004, who were identified as homeless or marginally housed. The ED is proximal to homeless communities and social housing complexes and is renowned for its care of people experiencing homelessness. Patient data were linked to national death records through January 2020 by the Australian Institute for Health and Welfare (AIHW).

### Homeless/marginal housing classification

Consistent with prior studies,^14^^,^^15^ patients whose medical records documented their accommodation during at least one ED presentation as ‘no fixed abode’ or as an address corresponding to emergency or transitional accommodation, a boarding house or public housing, were identified and the medical record further examined to confirm the level of homelessness and classify the housing status. This was completed by a member of the research team (GM) who was embedded in the hospital’s homeless service and who held extensive, first-hand knowledge of the local housing environment. Each presentation by an identified patient over the 2-year period was categorized. If patients moved between categories, their most common level of homelessness during 2003/04 was recorded. Details of this process are published.^16^^,^^17^ Chamberlain and Mackenzie’s definition was used to classify level of homelessness.^18^ Individuals experiencing primary, secondary or tertiary homelessness were classified as ‘homeless’. Those experiencing marginal housing were classified as ‘marginally housed’. See Supplementary Material (available as Supplementary data at *IJE* online) for definitions of housing classification, including inter-rater reliability data.

### Housed comparison group

A random sample of ED patients without a documented history of homelessness/marginal housing (i.e. ‘housed’), who presented at least once in 2003/04 (*n *= 39 528), was selected as a comparison group in a 3:1 ratio to the exposed group (housed *n* = 4725, marginally housed and homeless *n* = 1574).

### Ascertainment of mortality

The AIHW National Death Index (NDI) compiles death registrations in Australia. It contains person-level records, including facts [i.e. date of death and state/territory where death was registered, (through January 2020)] and cause(s) of death [i.e. up to 14 causes of death mapped to the International Statistical Classification of Diseases and Related Health Problems, 10th Revision (ICD-10) (through December 2018].^19^ Cause of death data are delayed due to time needed to map text fields to ICD-10 codes.

### Classification of mortality data

Cause of death was classified by ICD-10 chapter,^19^ consistent with prior research.^5^ Given the high prevalence of alcohol- and drug-related deaths among homeless populations,^3^^,^^8^ these deaths were examined in detail. Deaths due to suicide, drugs or alcohol were classified as per the Office of National Statistics, UK^20–22^ (see Supplementary Material).

The first listed cause of death was considered the primary cause. Exceptions were alcohol- and drug-related deaths, where both the primary cause (death due directly to alcohol or drugs) and the primary cause plus other causes listed were considered (alcohol and/or drugs contributing to the death).

### Statistical analysis

The cohort was described using frequencies/percentages and medians/interquartile ranges. Patient characteristics were compared using Fisher’s exact test for categorical variables and Wilcoxon rank sum or Kruskal–Wallis tests for continuous variables.

Given the epidemiological nature of our study and left truncation of the data, we chose age as the time scale.^23^ This is advantageous as age is effectively controlled within the analysis. We calculated age from date of birth to either the date of death or 31 January 2020, whichever came first. Kaplan–Meier (KM) survival analysis was performed to assess survival, with log-rank tests to assess significance (*P *<0.05).

Competing risks are a common occurrence in survival analysis and arise when a patient is at risk of more than one mutually exclusive event, such as death from different causes.^24^ To address competing risks, cause-specific hazards were modelled and transformed into cumulative incidence functions using the stpm2 and stpm2cif commands in Stata.^25^ Cumulative incidence functions (CIF) for deaths from external causes, cancer, CVD and respiratory diseases were created. All other deaths were grouped as ‘other’ and a CIF was developed. Stacked CIF graphs by housing status were created to allow for visualization of the total probability of death and the contribution of each cause to that probability.

An unadjusted Cox proportional hazards model estimated the associations between housing status index and all-cause and cause-specific mortality, including alcohol and drugs separately. Hazard ratios (HRs) and 95% confidence intervals (CIs) were generated for homeless and marginally housed patients, with housed patients as the reference category. We assessed for violations of the proportionality assumption by calculating Schoenfeld residuals. If proportionality did not hold, flexible non-proportional parametric models were created using restricted cubic splines to incorporate time-dependent effects.^25^ Statistical analyses were performed using Stata V17.0.

## Results

Data for 6290 unique patients were linked to national mortality data (99.9% linkage, Supplementary Figure S1, available as Supplementary data at *IJE* online). Homeless and marginally housed patients were more likely to be male, born in Australia and to identify as Aboriginal and/or Torres Strait Islander compared with housed patients (*P* < 0.001, Table 1).

**Table 1:** Characteristics of homeless, marginally housed and housed patients at the time of emergency department presentation in 2003/04

	Homeless			Marginally housed			Housed	
	(*n* = 1050)			(*n* = 518)			(*n* = 4722)	
	Median	IQR	*P*-value	Median	IQR	*P*-value	Median	IQR
Age at index presentation	38	28–50	<0.001	49	34–68	<0.001	40	27–62
Age at death	57	47–70	<0.001	73	57–83	<0.001	79	66–87
	*n*	%		*n*	%		*n*	%

Sex			<0.001			<0.001		
Male	828	78.9		345	66.6		2536	53.7
Female	222	21.1		173	33.4		2186	46.3
Born in Australia			<0.001			<0.001		
Yes	793	75.6		371	71.6		2696	57.1
No	256	24.4		147	28.4		2024	42.9
Aboriginal & Torres Strait Islander			<0.001			<0.001		
Yes	75	7.1		27	5.2		48	1.1
No	975	92.9		491	94.8		4674	98.9
English primary language			<0.001			<0.002		
Yes	1021	97.5		485	93.8		4200	89.3
No	26	2.5		32	6.2		503	10.7
Deceased			<0.001			<0.001		
Yes	382	36.4		259	50.0		1204	25.5
No	668	63.6		259	50.0		3518	74.5
Death registered in Victoria			<0.001			<0.01		
Yes	331	86.7		239	92.3		1159	96.3
No	51	13.3		20	7.7		45	3.7

*P*-values based on Fisher’s exact test.

IQR, interquartile range.

**Figure 1:**
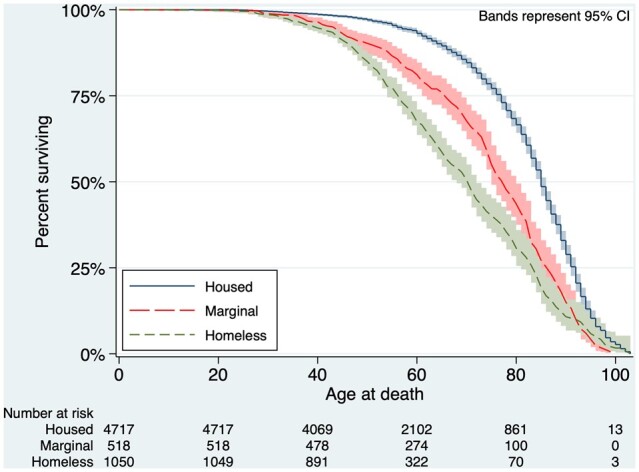
Kaplan–Meier survival curves for all-cause mortality by housing status over a 16-year period

The median age of death was younger in homeless (57 years) and marginally housed patients (73 years) compared with housed patients (79 years; *P* < 0.001). KM survival curves demonstrated a gradient in age at death by housing status (Figure 1).

Risk of all-cause mortality over the 16-year follow-up period was increased for homeless (HR = 4.0, 95% CI = 2.0–3.3) and marginally housed (HR = 2.6, 95% CI = 3.4–4.8) patients after their index (first) 2003/04 ED presentation, compared with housed patients who presented for ED care during the same period (Table 2).

**Table 2:** Estimated hazard ratios for all-cause and cause-specific deaths in homeless and marginally housed patients

	Cox HR	FPNP HR	95% CI	*P*-value
Housed (reference)	1			
**All-causes**				
Homeless		4.0	2.0–3.3	<0.0001
Marginally housed		2.6	3.4–4.8	<0.0001
**Cause of death**				
External				
Homeless	6.11		4.47–8.35	<0.0001
Marginally housed	3.57		2.36–5.40	<0.0001
Circulatory				
Homeless		4.92	2.78–8.70	<0.0001
Marginally housed		1.44	0.43–4.79	0.550
Cancer				
Homeless	1.55		1.15–2.09	0.004
Marginally housed	1.34		0.99–1.82	0.060
Respiratory				
Homeless		2.14	0.68–6.77	0.194
Marginally housed		4.69	1.82–12.05	<0.001
Homeless		4.09	2.78–6.03	<0.0001
Marginally housed		3.31	2.03–5.38	<0.0001
Alcohol (direct cause)				
Homeless	8.91		6.22–12.78	<0.0001
Marginally housed	4.52		2.87–7.13	<0.0001
Drugs (direct cause)				
Homeless	7.75		5.04–11.89	<0.0001
Marginally housed	4.21		2.35–7.54	<0.0001

HR, hazard ratio; FPNP, flexible non-proportional parametric hazards model; CI, confidence interval.

a Diseases of the (i) eye and adnexa, (ii) ear and mastoid process, (iii) skin and subcutaneous tissue, (iv) musculoskeletal system and connective tissue, (v) genitourinary system, (vi) blood and blood-forming organs and certain disorders involving the immune system, (vii) endocrine, nutritional and metabolic system, (viii) nervous system; and (ix) deaths due mental, behavioural and neurodevelopmental disorders; and symptoms, signs and abnormal clinical and laboratory findings, not elsewhere classified.

**Figure 2:**
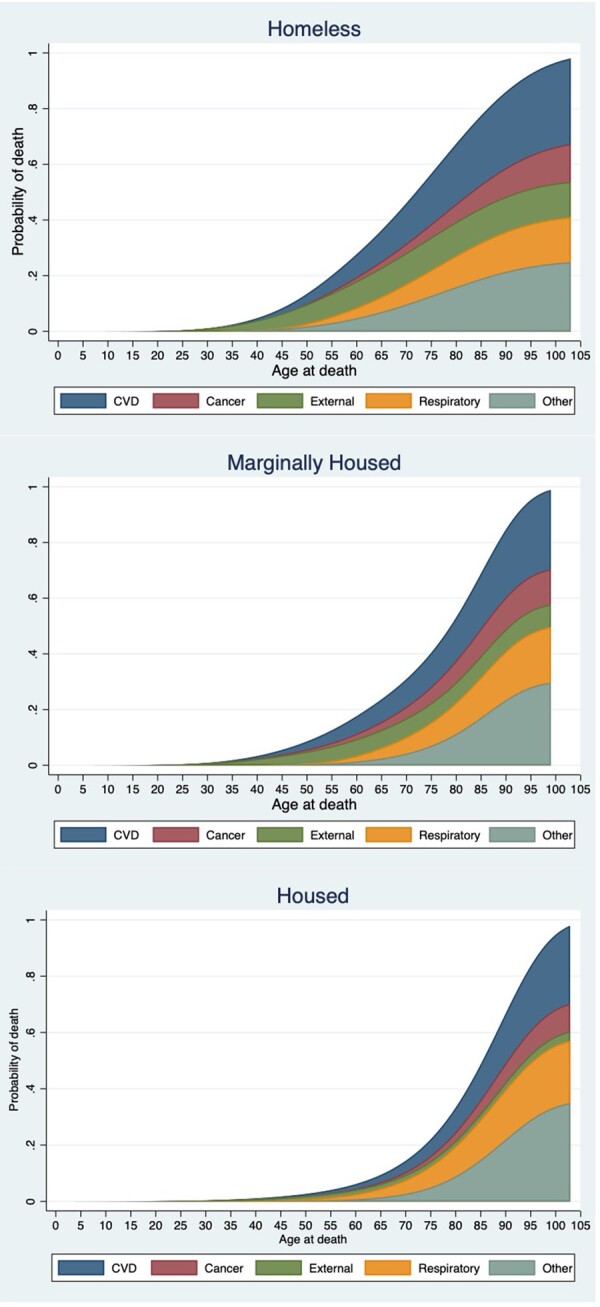
Stacked cumulative incidence functions for cause-specific deaths by housing status in patients attending an emergency department in 2003/04. CVD, cardiovascular disease

The stacked CIF visualizes the total probability of death by age and the contribution each cause makes to this probability (Figure 2). These graphs illustrate the magnitude of mortality differences between the three groups. The curves were left-shifted for both homeless and marginally housed patients relative to housed patients, demonstrating that death begins at younger age and the death rate rises earlier. As an example, the probability of dying by 55 years for a homeless patient is 0.2, whereas it is 0.1 and 0.02 for a marginally housed and housed patient, respectively. The role each cause of death played over time also differed. In contrast to housed patients, the persistent bandwidth associated with external causes among homeless and marginally housed patients indicates that the probability of death from an external cause continues, regardless of age. The probability of dying only starts to equalize between the three groups at 95 years.

The leading causes of death among homeless patients were external (24.8%), CVD (21.3%) and cancer (14.7%), and among marginally housed patients they were CVD (25.9%), cancer (19.1%) and external causes and respiratory diseases (both 12.8%; Table 3).

**Table 3:** Leading cause of death among homeless, marginally housed and housed patients by ICD-10 chapter

	Homeless				Marginally housed				Housed			
	(*n* = 1050)				(*n* = 518)				(*n* = 4722)			
	Deceased (*n* = 347)				Deceased (*n* = 251)				Deceased (*n* = 1136)			
ICD-10 Chapter	*n*	%	Age at death	IQR	*n*	%	Age at death	IQR	*n*	%	Age at death	IQR
Infections	9	2.6	60	48–64	8	3.2	78	76–83	30	2.6	74	61–87
Cancers	51	14.7	61	54–72	48	19.1	75	65–83	293	25.8	73	64–83
Other (e.g. prostate, breast)	19	5.5			18	7.2			120	10.6		
Digestive	15	4.3			14	5.6			88	7.8		
Lung	16	4.6			12	4.8			57	5.0		
Lymph/blood	1	0.3			4	1.6			28	2.5		
Endocrine, nutritional and metabolic	22	6.3	79	70–86	16	6.4	77	62–86	61	5.4	79	70–86
Mental, behavioural and neurodevelopmental disorders	24	6.9	72	54–83	12	4.8	77	75–88	44	3.9	87	81–91
Nervous system	7	2.0	56	42–85	9	3.6	73	57–89	52	4.6	83	72–91
Circulatory system	74	21.3	64	54–75	65	25.9	81	74–87	353	31.1	83	74–89
Stroke	6	1.7			7	2.8			35	3.1		
Ischaemic heart disease	38	11.0			31	12.5			158	13.9		
Other (e.g. intracerebral haemorrhage)	12	3.5			16	6.4			90	7.9		
Other heart disease (e.g. congestive heart failure)	18	5.2			11	4.3			70	6.2		
Respiratory system	31	8.9	74	59–80	32	12.8	70	61–81	107	9.4	82	72–87
COPD	16	4.6			22	8.8			49	4.3		
Other (e.g., pneumonia)	15	4.3			10	4.0			58	5.1		
Digestive system	30	8.7	52	45–60	17	6.8	55	45–62	53	4.7	74	60–85
Liver	26	7.5			14	5.6			17	1.5		
Other (e.g. gastrointestinal haemorrhage)	4	1.2			3	1.2			36	3.2		
External causes	86	24.8	44	35–51	32	12.8	42	37–57	75	6.6	41	32–56
Accidents	64	18.4			20	8.0			48	4.2		
Self-harm	13	3.8			5	2.0			18	1.6		
Other (e.g. assault, poisoning of undetermined intent)	9	2.6			7	2.7			9	0.8		
Other^a^	13	3.8	62	50–74	12	4.8	68.5	46–80	68	6.0	76	60–88

COPD, chronic obstructive pulmonary disease; ICD-10, International Statistical Classification of Diseases and Related Health Problems, 10th Revision; IQR, interquartile range.

a Diseases of the (i) eye and adnexa, (ii) ear and mastoid process, (iii) skin and subcutaneous tissue, (iv) musculoskeletal system and connective tissue, (v) genitourinary system, (vi) blood and blood-forming organs and certain disorders involving the immune mechanism; and symptoms, signs and abnormal clinical and laboratory findings, not elsewhere classified.

Among homeless and marginally housed patients 10.1% and 5.6% of deaths, respectively, were due to alcohol, compared with 1.3% in housed patients (Table 4). Considerably more deaths due to drugs were reported among homeless (15.9%) and marginally housed (6.8%) patients relative to housed patients (3.0%). Considering deaths in which alcohol and/or drugs were involved (i.e. primary cause or contributor), alcohol was recorded in 21.3% and 11.7% of deaths among homeless and marginally housed patients, respectively, compared with 4.5% in housed patients. Drugs contributed to 19.9% of deaths among homeless patients and 7.2% in marginally housed patients, compared with 3.5% in housed patients.

**Table 4:** Deaths related to alcohol, drugs and suicide by housing status in 2003/04

	Homeless				Marginally housed				Housed			
	(*n* = 347)				(*n* = 251)				(*n* = 1136)			
Direct cause of death	*n*	%	Age at death	IQR	*n*	%	Age at death	IQR	*n*	%	Age at death	IQR
Alcohol	35	10.1	53	48–60	14	5.6	56	45–67	15	1.3	62	55–72
Drugs	55	15.9	44	35–51	17	6.8	37	29–41	34	3.0	37	33–46
Suicide^a^	20	5.8	38	33–48	9	3.6	38	36–49	24	2.1	39	30–51
Contributed to death^b^												
Alcohol	74	21.3			29	11.7			51	4.5		
Drugs	69	19.9			18	7.2			40	3.5		

ICD-10, International Statistical Classification of Diseases and Related Health Problems, 10th Revision; IQR, interquartile range.

a Coding for suicide includes deaths of undetermined intent, unlike ICD-10 chapter coding which categorizes self-harm only when the intent is known.

b Not mutually exclusive (e.g. a patient whose death was classified as an illicit drug overdose while intoxicated by alcohol was counted twice).

Regarding cause-specific hazards (Table 2), homeless patients had an excess risk of death due to external causes (HR = 6.1, 95% CI = 4.47–8.35), CVD (HR = 4.9, 95% CI = 2.78–8.70), cancer (HR = 1.5, 95% CI = 1.15–2.09) and other causes (HR = 4.1, 95% CI = 2.78–6.03) compared with housed patients. Marginally housed patients had a higher risk of death for all causes, except CVD and cancer, relative to housed patients. The HRs of death from external causes, respiratory diseases and other causes were 3.6 (95% CI = 2.36–5.40), 4.7 (95% CI = 1.82–12.05) and 3.3 (95% CI = 2.03–5.38) times higher than housed patients, respectively. When risk of death from alcohol or drugs was considered, the risk of death due to alcohol was greater in homeless (HR = 8.9, 95% CI = 6.22–12.78) than in marginally housed patients (HR = 4.5, 95% CI = 2.87–7.13). This was replicated in deaths due to drugs, with HRs of 7.6 (95% CI = 5.04–11.89) and 4.2 (95% CI = 2.35–7.54) in homeless and marginally housed patients, respectively.

## Discussion

This study compared 16-year mortality patterns among homeless, marginally housed and housed adults who presented at a metropolitan ED in Melbourne, Australia. It is the first study on mortality and causes of death to include homeless and marginally housed patients over an extended follow-up period, using national mortality data. The risk of premature mortality was four times higher in patients who were homeless relative to those who were housed, similar to international findings.^8^^,^^9^ Compared with housed patients, marginally housed patient were at 2.6 times greater risk of premature mortality.

The leading causes of death among homeless patients were external causes (e.g. accidents, suicide), CVD and cancer, replicating international studies.^4^^,^^9^^,^^26^ The leading causes of death among marginally housed patients were similar to those among housed patients, with cardiovascular, respiratory diseases and cancer being the most common causes of death in both groups. The exception was external causes, which was the third most common cause of death among marginally housed patients, suggesting some cause-of-death similarity between homeless and marginally housed patients.

Taking account of competing risk, marked inequality in the probability of death was observed between the three groups at similar ages. To contextualize this finding, the probability of a homeless patient in this study dying at age 55 years would be equivalent to a male dying at age 72 and a female at age 77 in the general population in Australia.^27^ This comparison among marginally housed patients reveals a less confronting, yet still drastic, difference: a 55-year-old marginally housed person in this study has the same probability of dying as a 62-year-old male and a 70-year-old female in the general population.^27^ It could be argued that comparing data from ED patients with those from the general population is inappropriate, as the variation in probability figures could be akin to comparing people who are unwell with those in good health; however, given that housed patients’ probability of death by 55 years was similar to the Australian general population,^27^ this does not appear to be the case in this study.

External causes accounted for nearly 25% of deaths in homeless patients and 12% in marginally housed patients. Comparable studies conducted in England and Canada found similar rates of deaths due to external causes among patients who were homeless (22–27%).^5^^,^^7^ When compared with housed patients, marginally housed patients were 3.5 times as likely to die from an external cause, whereas homeless patients were more than six times as likely. Given that a substantial proportion of deaths by external causes were substance-related, further analyses were undertaken.

Deaths due to alcohol consumption included both acute (e.g. alcohol poisoning) and chronic effects (e.g. alcoholic liver disease) and were more common in homeless patients (10%) than in marginally housed (5.6%) and housed patients (1.3%). Finding elevated alcohol-related deaths among homeless patients was not surprising, given that alcohol use disorders are the most common diagnoses among people who experience homelessness.^28^ In terms of cause-specific risk, deaths among homeless and marginally housed patients were 8.9 and 4.5 times more likely to be alcohol-related than among those who were housed, respectively. The rates of alcohol-related deaths among people with a documented or current history of homelessness varies considerably in the literature (<1–17%),^5^^,^^8^ making direct comparison difficult. Although our findings align with those reported in prior studies, it is likely our figures underestimate the true number of deaths attributable to alcohol, such as those due to cancer (e.g. digestive tract) or non-alcohol specific CVD, as these are omitted from the applied definitions used in this study. Indeed, the risks of homeless patients dying from CVD and cancer were 4.9 and 1.4 times those of housed patients, respectively; alcohol consumption may have contributed to these deaths.

In the current study, the proportion of deaths directly caused by drugs was higher than that due to alcohol across all three groups. Similar rates of death due to drug overdose have been reported in homeless populations in the USA (e.g. adults who sought services at Boston Health Care for the Homeless Program, 2003–08; 16.8%).^9^ Of note, the applied definition for drug-related deaths used in this study only considered deaths from poisoning or a drug-induced mental disorder. As such, other deaths that may have resulted from illnesses that could be exacerbated by chronic drug use (e.g. hepatitis C) are not included in these statistics. The risk of drug-associated death was 4.2 and 7.8 times higher among those who were identified as marginally housed or homeless, respectively, than among housed patients.

Death due to CVD was common to all three groups in this study, but homeless patients’ median age at death from CVD was almost 20 years lower than the corresponding ages for marginally housed and housed patients (64 years, 81 years and 83 years, respectively). Furthermore, the risk of a patient with a history of homelessness dying from CVD was almost five times that of a housed patient. A recent systematic review found the risk of premature mortality from CVD in people experiencing homelessness was three times that in housed populations.^29^ This figure is lower than our findings and may be due to the broader definition of homelessness used by Al-Shakarchi *et al*., coupled with our focus on patients who presented for ED care (as opposed to the broader homeless population) who may have poorer health. Al-Shakarchi *et al*.^29^ noted that unique risk factors more common among homeless people (e.g. nutritional deficiencies, barriers to health care, high smoking prevalence) occur in addition to typical risk factors (e.g. hypertension, diabetes). Collectively, these yield a high CVD burden among people who experience homelessness.^29^

We found similar risk for a CVD death in marginally housed and housed patients. It is possible that the unique challenges that contribute to the high burden of CVD among homeless patients may be lower among marginally housed individuals. Indeed, prior research has shown that moving into housing is associated with significant reductions in use of emergency medical services.^30^ For individuals with illness or disability, housing may facilitate more effective management of disease. For instance, individuals residing in marginal housing may have fewer safety concerns than homeless people (e.g. victimization while rough sleeping) and be better able to prioritize health-promoting behaviour.^31^

Death from respiratory disease was more common in marginally housed patients; with a higher incidence of chronic obstructive pulmonary disease (COPD), they were 4.7 times as likely to die from respiratory disease as housed patients. It is possible that those who are marginally housed are at greater risk of respiratory disease given its higher prevalence in poorer socioeconomic areas^32^ and documented association with poor housing conditions.^12^^,^^33^^,^^34^ Additionally, in Australia people who require accommodation and have a diagnosis of COPD (or any condition that results in disability) are more likely to be marginally housed, as people with disability are prioritized for social housing.^35^

Previous research has reported findings similar to those in the present study of higher than anticipated mortality in formerly homeless people.^36^ Henwood *et al*.^36^ found individuals identified as having medical conditions (e.g. cancer) were prioritized for placement in housing programmes, which contributed to an elevated mortality rate among people who were previously homeless. It is possible that a similar situation has occurred in this study with respect to respiratory disease. It is also conceivable that housing is unable to alter the trajectory of advanced conditions among adults who have a history of chronic homelessness and long-standing health problems.^37^

These findings indicate that being marginally housed may assuage some risks of premature mortality associated with homelessness; however, marginal housing is not equivalent to being appropriately and stably housed. The mortality disparity between marginally housed and housed patients in this study may be the consequence of health inequalities associated with low income, the sequelae of previous homelessness, as suggested by Tsai *et al.*,^37^ or a combination of these or other factors. Nevertheless, the results of this study support the provision of housing as an intervention that may reduce premature mortality.

Strengths of this study include the detailed examination of medical records to determine housing status, a high number of cases and the use of national mortality data. Sophisticated analysis to ascertain the risk of mortality by housing status, moving beyond standard Cox regression to apply flexible modelling that incorporated time-dependent effects, enabled survival analysis over 16 years—a first in literature on mortality among people who experience homelessness. Similar mortality outcomes reported in the present and international studies add to the evidence documenting housing as a social determinant of health and advances the literature by demonstrating similar findings in a sample of marginally housed patients. Further, detailing all-cause and cause-specific mortality in patients who are at risk of homelessness fills a gap in the literature and is a novel contribution of this study.

Several limitations warrant mention. Individuals who were homeless were identified via medical record chart review. Underestimation of homelessness/marginal housing may have occurred due to a reliance on patient self-report of usual accommodation. Although this challenge is inherent when using routinely collected health data, it was mitigated by skilled examination of individual medical records by a member of the research team. Additionally, the definition used for marginal housing, commonly used in the Australian context, may not be transferable to other national contexts.

Ascertainment of homelessness occurred over a 24-month period and housing trajectories were not tracked thereafter. Thus, we do not have data on whether patients’ housing status changed or remained the same over 2005–20. It is possible that individuals who exit homelessness have reduced mortality rates, and the possibility that mortality among that group was overestimated should be considered. Alternatively, the accommodation status of marginally housed and housed patients may have deteriorated following the study enrolment period, potentially leading to mortality associated with housing status to be underestimated in those groups. Deaths due to cardiovascular and respiratory diseases and cancer among homeless and marginally housed patients may be explained, in part, by smoking. The prevalence of smoking in homeless populations ranges between 57% and 82% and is well above that of general populations.^38^ Unfortunately, lack of ICD-10 codes to capture this health risk behaviour precluded consideration of this factor in our analyses. Finally, we were unable to account for pre-existing conditions that may have contributed to patients’ morbidity prior to the study. Future research should attempt to document morbidity prior to changes in housing status, to understand how shifts in housing status improve or degrade health.

## Conclusion

This is the first study to move beyond all-cause mortality to quantify premature mortality risk and causes of death among ED patients who were homeless and marginally housed. This long-term analysis demonstrates persistent health inequalities among housed, marginally housed and homeless patients. Compared with patients who sought ED care who were housed, the homeless and marginally housed patients died 22 and 6 years younger on average, respectively. Our findings suggest that being homeless or marginally housed at a single point in time is a risk factor for long-term premature mortality.

## Ethics approval

Approval was obtained from St Vincent’s Hospital, Melbourne (HREC/18/SVHM/236) and the Australian Institute for Health and Welfare (EO2019/3/1072) human research ethics committees.

## Supplementary Material

dyad006_Supplementary_Data

## Data Availability

The data underlying this article were provided by St Vincent’s Hospital, Melbourne, and the Australian Institute of Health and Welfare, by permission. Data will be shared on request to the corresponding author with permission of St Vincent’s Hospital, Melbourne, and the Australian Institute of Health and Welfare.
