# Supplementary material for: Premature mortality 16 years after emergency department presentation among homeless and at risk of homelessness adults: a retrospective longitudinal cohort study
Source: Int J Epidemiol. 2023 Feb 8;52(2):501–11. doi: 10.1093/ije/dyad006 (PMC10114125; doi:10.1093/ije/dyad006)
Supplement: dyad006_Supplementary_Data [file dyad006_supplementary_data.docx]

**SUPPLEMENTARY MATERIAL**

**Housing classification**

For each ED presentation at the metropolitan hospital in Melbourne, Australia, information about housing status was captured in the patient administration system by the variable “usual accommodation.” To ensure the accuracy of this variable, medical records of patients identified as being homeless were examined in detail to confirm their housing status and to classify level of homelessness according to Chamberlain and Mackenzie.^1^

Chamberlain and Mackenzie’s^1^ definition outlines four types of homelessness in Australia: i) primary (without conventional accommodation; e.g., living on the streets, sleeping in parks, squatting in derelict buildings); ii) secondary (moving between various forms of temporary shelters, including emergency or transitional accommodation [e.g., youth refuges, hostels], other households [e.g., friends], and boarding houses on a short-term basis [12 weeks or less]); iii) tertiary (living in a single room in a private boarding house on a medium- to long-term basis without a separate bedroom and living room, private bathroom and kitchen, or security of tenure); and iv) marginal housing (housing situation close to the minimum standard, including public housing requiring rental assistance or other unstable rental arrangement).^1^

Three abstractors collated data for patients who were homeless. Training was conducted over two days and review of abstractor accuracy was performed daily. One abstractor took the position of leader in the data collection process (author initials removed for peer review) and examined 80% of all data collected. All abstractors were masked to the aetiologic relationships being investigated in the study.

At the outset of data abstraction, the abstractors conducted an independent review of the first 20 medical records and inter-rater reliability was determined. Agreement for the category homeless or not homeless was 96.7% across the three abstractors. Agreement for the level of homelessness was 73.3% for the three abstractors. In 12 of the 20 cases, all three abstractors agreed with the four categories. With the other eight cases, two of the three abstractors agreed.^2^

**Housed comparison group**

Matching unhoused/marginally housed to housed individuals was considered for this study; however, it was decided that applying Cox-regression models would be more powerful, as previously shown in the literature.^3^ Therefore, a random sample of patients without a documented history of homelessness/marginally housing, who presented at least once to the ED in 2003/04 (n=39,528) was selected in a 3:1 ratio of housed to unhoused/marginally housed patients.

**Table S1. Categorisation of deaths by ICD-10 chapter and codes**

| **ICD chapter** | **ICD-10 codes** |
| --- | --- |
| Infectious and parasitic diseases | A00–B99 |
| Cancers | C00–D48 |
| Diseases of blood, blood-forming organs, and certain disorders involving the immune mechanism | D50–D89 |
| Endocrine, nutritional and metabolic diseases | E00–E90 |
| Mental and behavioural disorders | F00–F99 |
| Diseases of the nervous system | G00–G99 |
| Diseases of the eye and adnexa | H00–H59 |
| Diseases of ear and mastoid process | H60–H95 |
| Diseases of the circulatory system | I00–I99 |
| Diseases of the respiratory system | J00–J99 |
| Diseases of the digestive system | K00–K93 |
| Diseases of the skin and subcutaneous tissue | L00–L99 |
| Diseases of the musculoskeletal system and connective tissue | M00–M99 |
| Diseases of the genitourinary system | N00–N99 |
| Congenital malformations, deformations and chromosomal abnormalities | Q00–Q99 |
| Symptoms, signs & abnormal clinical and laboratory findings, not elsewhere classified | R00–R99 |
| Injury, poisoning and certain other consequences of external causes | S00–T98 |
| External causes of morbidity and mortality | V01–Y98 |
| Factors influencing health status and contact with health services | Z00–Z99 |

ICD-10= International Statistical Classification of Diseases and Related Health Problems-10th Revision

**Table S2. Definitions for deaths due to suicide, alcohol, and drugs and associated ICD-10 codes**

| **Cause of death** | **ICD-10 codes** |
| --- | --- |
| **Suicide** deaths were those where the underlying cause was intentional self-harm or where the underlying cause was event of undetermined intent for those aged 15 years and older.^4^ | X60-X84, Y10-Y34, X610, X620, X650, X678, X690, X700, X702, X709, X768, X780, X800, X803, X804, X808, X818, X910, X918, X999 |
| **Alcohol-related deaths** include conditions where death was a direct consequence of alcohol misuse, including chronic conditions associated with continued misuse of alcohol and acute alcohol-related conditions.^5^ | E24.4, G31.2, G62.1, G72.1, I42.6, K29.2, K85.2, K86.0, Q86.0, R78.0, F10, K70, X45, F14, X65, Y15 |
| **Drug-related deaths** include accidents, suicides, and assaults involving drug poisoning, and deaths from drug abuse/dependence. This category does not include other adverse drug events (e.g., anaphylactic shock) or transport accidents where the driver was under the influence of drugs.^6^ | X40-X44, X60-X64, F11-F19 X85, Y10-Y14 |

ICD-10= International Statistical Classification of Diseases and Related Health Problems-10th Revision

**Figure S1. Data flow for linkage**

**Patients aged 18 or older who presented for emergency department care in 2003/04**

N=40,978

**Linkage to Australian Institute of Health and Welfare National Death Index**

n=6,290

Duplicate records n=7

Not matched n=2

**Housed (n=4722)**

Facts of death n=1,204 (26%)

Cause of death n=1,136 (24%)

**Homeless (n=1050)**

Facts of death n=382 (36%)

Cause of death n=347 (33%)

**Marginally Housed (n=518)**

Facts of death n=259 (50%)

Cause of death n=251 (48%)

**Homeless**

Identified as experiencing primary, secondary, or tertiary homelessness

n=1,056

**Marginally Housed**

Identified as experiencing marginal housing

n=518

**Housed**

No indication of primary, secondary, or tertiary homelessness; nor marginal housing

n=4,725

Facts of death data n=1,845

Cause of death data n=1,734

**References**

1. Chamberlain C, Mackenzie D. Understanding Contemporary Homelessness: Issues of Definition and Meaning. *Aust J Soc Issues* 1992; **27**: 274-97.

2. Moore G, Gerdtz M, Manias E, Hepworth G, Dent A. Socio-demographic and clinical characteristics of re-presentation to an Australian inner-city emergency department: implications for service delivery. *BMC Public Health* 2007; **7**: 320.

3. Brazauskas R, Logan BR. Observational Studies: Matching or Regression? *Biol Blood Marrow Transplant* 2016; **22**: 557-63.

4. Office of National Statistics. *Suicide rates in the UK, Quality and Methodology Information*. <https://www.ons.gov.uk/peoplepopulationandcommunity/birthsdeathsandmarriages/deaths/methodologies/userguidetomortalitystatisticsjuly2017#cause-of-death-coding>; (9 November 2022, date last accessed).

5. Office of National Statistics. *Alcohol-specific deaths in the UK: registered in 2019*. <https://www.ons.gov.uk/peoplepopulationandcommunity/healthandsocialcare/causesofdeath/methodologies/alcoholrelateddeathsintheukqmi#concepts-and-definitions>; (9 November 2022, date last accessed).

6. Office of National Statistics. *Deaths related to drug poisoning in England and Wales QMI*. <https://www.ons.gov.uk/peoplepopulationandcommunity/birthsdeathsandmarriages/deaths/methodologies/deathsrelatedtodrugpoisoninginenglandandwalesqmi>; (9 November 2022, date last accessed).
